# The effects of BPA-BNCT on normal bone: determination of the CBE value in mice^‡^

**DOI:** 10.1093/jrr/rrad054

**Published:** 2023-07-29

**Authors:** Ryota Iwasaki, Ryutaro Yoshikawa, Ryo Umeno, Azusa Seki, Takehisa Matsukawa, Satoshi Takeno, Kazuhito Yokoyama, Takashi Mori, Minoru Suzuki, Koji Ono

**Affiliations:** Department of Veterinary Medicine, Gifu University, 1-1 Yanagido, Gifu-shi, Gifu 501-1193, Japan; Department of Veterinary Medicine, Gifu University, 1-1 Yanagido, Gifu-shi, Gifu 501-1193, Japan; Department of Veterinary Medicine, Gifu University, 1-1 Yanagido, Gifu-shi, Gifu 501-1193, Japan; HAMRI Co. Ltd., 2638-2 Ozaki, Koga-shi, Ibaragi 306-0101, Japan; Department of Epidemiology and Environmental Health, Juntendo University, 2-1-1 Hongo, Bunkyo-ku, Tokyo 113-8421, Japan; Department of Forensic Medicine, Juntendo University, 2-1-1 Hongo, Bunkyo-ku, Tokyo 113-8421, Japan; Department of Radiation Oncology, Osaka Medical and Pharmaceutical University, 2-7 Daigaku-machi Takatsuki-shi, Osaka 569-8686, Japan; Kansai BNCT Medical Center, Osaka Medical and Pharmaceutical University, 2-7 Daigaku-machi Takatsuki-shi, Osaka 569-8686, Japan; Department of Epidemiology and Environmental Health, Juntendo University, 2-1-1 Hongo, Bunkyo-ku, Tokyo 113-8421, Japan; Department of Epidemiology and Social Medicine, International University of Health and Welfare, 4-1-26 Akasaka, Minato-ku, Tokyo 107-8402, Japan; Department of Veterinary Medicine, Gifu University, 1-1 Yanagido, Gifu-shi, Gifu 501-1193, Japan; Particle Radiation Oncology Research Center, Institute for Integrated Radiation and Nuclear Science, Kyoto University, 2-1010, Asashiro-nishi, Kumatori-cho, Sennan-gun, Osaka 590-0494, Japan; Kansai BNCT Medical Center, Osaka Medical and Pharmaceutical University, 2-7 Daigaku-machi Takatsuki-shi, Osaka 569-8686, Japan

**Keywords:** bending strength, bone, boron neutron capture therapy, BPA, CBE factor, distribution

## Abstract

Boron neutron capture therapy (BNCT) with p-boronophenylalanine (BPA) is expected to have less effect on the decrease in normal bone strength than X-ray therapy. However, the compound biological effectiveness (CBE) value necessary to convert the boron neutron capture reaction (BNCR) dose into a bioequivalent X-ray dose has not been determined yet. The purpose of this study was to evaluate the influence of BNCT on normal bone in mice and to elucidate the CBE factor. We first searched the distribution of BPA in the normal bone of C3H/He mice and then measured the changes in bone strength after irradiation. The CBE value was determined when the decrease in bone strength was set as an index of the BNCT effect. The ^10^B concentrations in the tibia after subcutaneous injection of 125, 250 and 500 mg/kg BPA were measured by prompt gamma-ray spectroscopy and inductively coupled plasma (ICP)-atomic emission spectrometry. The ^10^B mapping in the tibia was examined by alpha-track autoradiography and laser ablation-ICP-mass spectrometry. The ^10^B concentration increased dose-dependently; moreover, the concentrations were maintained until 120 min after BPA administration. The administered ^10^B in the tibia was abundantly accumulated in the growth cartilage, trabecular bone and bone marrow. The bone strength was analyzed by a three-point bending test 12 weeks after irradiation. The bending strength of the tibia decreased dose-dependently after the irradiation of X-ray, neutron and BNCR. The CBE factor was obtained as 2.27 by comparing these dose-effect curves; the value determined in this study will enable an accurate dosimetry of normal bone.

## INTRODUCTION

The normal bone is often included in the irradiated field when performing conventional radiotherapy with X-rays. It has been reported that bone fracture due to decreased bone strength and osteonecrosis can occur as late effects in the pelvic bones, ribs, limbs, vertebrae and jaw bones [1–6]. The following irreversible mechanisms causing the decrease in bone strength have been recognized: (i) changes in the bone structure such as the decrease in bone volume; (ii) reduction in bone mineral density and (iii) changes in the extracellular matrix, such as increased collagen cross-linking ratio and the formation of glycation end products [7–10].

On the other hand, boron neutron capture therapy (BNCT) is expected to reduce the late effects on normal bone compared with X-ray, considering its anti-tumor mechanism: BNCT is a radiotherapy that destroys the tumor cells with α-particle and the ^7^Li-nucleus, produced through the nuclear reaction between the administered boron-10 (^10^B), which tends to accumulate in the tumor cells and thermal neutrons. The ranges of these particles are ≤10 μm, which does not exceed the diameter of an ordinary cell, and their effect is almost fully restricted to cells that have taken up the boron drug. These factors, together with the selective accumulation of the boron drug in tumor cells, allow the selective destruction of tumor cells and reduce the damage to normal tissues [11]. However, despite the tumor cell selectivity of ^10^B accumulation, there is a significant accumulation of the boron drug in normal tissues as well [12]. As a result, adverse events such as dermatitis, mucositis and brain necrosis similar to those observed after X-ray therapy have been reported to occur in skin, mucosa and brain tissues [11, 13, 14]. Although BNCT may cause late effects similar to X-rays or specific to BNCT in normal bones as well, its influence has not been evaluated.

In BNCT, most doses given to the tissue consist of doses from the neutron capture reactions of ^10^B-nucleus (boron neutron dose), those of ^14^N-nucleus (nitrogen dose), elastic collision between hydrogen nuclei and neutrons (hydrogen dose), secondary γ-rays associated with these reactions and primary γ-rays from neutron sources. The latter three can be converted to biological X-ray equivalent doses by multiplying the physical doses by a constant relative biological effectiveness (RBE) value because the content and distribution of nitrogen and hydrogen do not differ between tissue types. However, the conversion factor for the boron neutron doses varies depending on the combination of the type of boron compound, target tissue and observed adverse event because the distribution of boron agents at the cellular level and cell types involved in adverse events differ for each tissue. There is no other way to determine this compound biological effectiveness (CBE) factor except through experimental studies, and this value as an indicator of late effect in normal bone has not yet been determined.

The objectives of this study were to analyze the uptake and distribution of p-boronophenylalanine (BPA) in normal bones of mice and to evaluate the influence of BNCT on their tibiae. Further, the CBE value of BPA was also determined when the fracture, a late effect that is a problem in clinical practice, was set as an indicator.

## MATERIALS AND METHODS

### Chemicals

The ^10^B-enriched boron compound BPA was obtained from Interpharma Praha, a.s. (Prague, Czech Republic). BPA was suspended in distilled water with fructose and 1 N NaOH to improve the solubility, then the mixture was stirred until it was completely dissolved [15]. Subsequently, the solution was adjusted the pH to 7.6 with 1 N HCl and was sterilized using a 0.22-μm filter. The final BPA concentration was 30 mg/ml (1.44 mg ^10^B/ml).

### Experimental animals

According to the previous study that established a murine model for radiation-induced bone fragility, female 8-week-old C3H/He mice were obtained from a specific pathogen-free colony at Japan SLC, Inc (Shizuoka, Japan) for all experiments [16]. To reduce the stress of transportation and allow the animals to adapt to the new environment, an acclimatization period of 1 week was considered. All of the animal experiments were approved by the Animal Research Committee of Gifu University and Institute for Integrated Radiation and Nuclear Science, Kyoto University (KURNS) and were conducted in accordance with the regulations of the Animal Care and Use Committee of Gifu University and KURNS.

### Boron concentration

BPA solutions at 125, 250 or 500 mg/kg body weight were administered subcutaneously. After blood samples were collected by cardiac puncture under 2–3% isoflurane anesthesia at 30, 60, 90 or 120 min after administration, mice were euthanized using CO_2_ gas and bilateral tibiae were immediately sampled (five mice for each BPA dose and time point). The ^10^B concentrations in the blood and tibia were measured by the following two methods.

#### Prompt gamma-ray analysis

Each sample was measured using a thermal neutron guide tube installed at KURNS [17]. The measured values for the tibia, which was the hard tissue, were corrected based on the elemental composition and material density values in ICRU REPORT 44 & 46 as follows: 


\begin{equation*}C_{{\text{Adj}}}=C_{\text{Meas}} \times{EC}_{{\text{Bone}}}/EC_{\text{Water}}\times D, \end{equation*}

where *C*_Adj_ and *C*_Meas_ are the corrected and measured ^10^B concentrations (ppm), respectively; *EC*_Bone_ and *EC*_Water_ are the compositions of hydrogen in bone and water, respectively (%); and *D* is the bone density (g/cm^3^).

#### Inductively coupled plasma atomic emission spectrometry

Each sample was digested by heating in nitric acid (60%) at 115°C for 15 min, then diluted with distilled water and divided into three test tubes. After measuring the boron concentration in these tubes using ULTIMA2 (HORIBA, Ltd, Kyoto, Japan), their average was taken and expressed in ppm.

### Visualization of ^10^B biodistribution in the tibia

Sixty minutes after administration of BPA at 125, 250 or 500 mg/kg, mice were euthanized, and their tibiae were collected (three mice for each BPA dose). Samples were prepared into 5-μm thick undecalcified serial sections, and ^10^B was visualized by the following two methods [18].

#### Autoradiography

Sections were pasted on a solid-state nuclear track detector, CR-39 (BRYOTRACK, Nagase-Landauer, Ltd, Ibaraki, Japan) and were irradiated with thermal neutrons at KURNS at fluences of 6.32 × 10^11^–1.36 × 10^12^ n/cm^2^ for 7–15 min. Subsequently, irradiated CR-39 was etched with PEW solution (15 wt% KOH + 65 wt% EtOH +20 wt% H_2_O) at 50°C for 8 min. By precisely superimposing the tracks on CR-39 caused by α-particle and 7Li-nucleus with the tissue section images, the distribution of the tracks in normal bone was observed under an optical microscope [19].

#### Laser ablation ICP mass spectrometry

The experiment was performed following the method of Tsurubuchi *et al*. using the NWR213 (Electro Scientific Industries, Inc, Portland, OR, USA) for sample introduction by laser ablation and Agilent 8800 (Agilent Technologies Japan) for ICP-MS [20]. The measurement conditions for laser ablation were as follows: wavelength, 213 nm; laser shape, square; laser size, 50 × 50 μm; intensity, 1.7 J/cm^2^; scan speed, 50 μm/s; and pulse ratio, 20 /s. The ICP-MS conditions were as follows: RF power, 1600 W; and Ar flow, 15.0 l/min. High-resolution analyses confined to the diaphysis were performed at a laser size of 10 × 10 μm and a scan speed of 7 μm/s. Boron localization was then visualized by semi-quantitative imaging based on the boron concentration obtained from prompt gamma-ray analysis (PGA).

### Irradiation

**Fig. 1:**
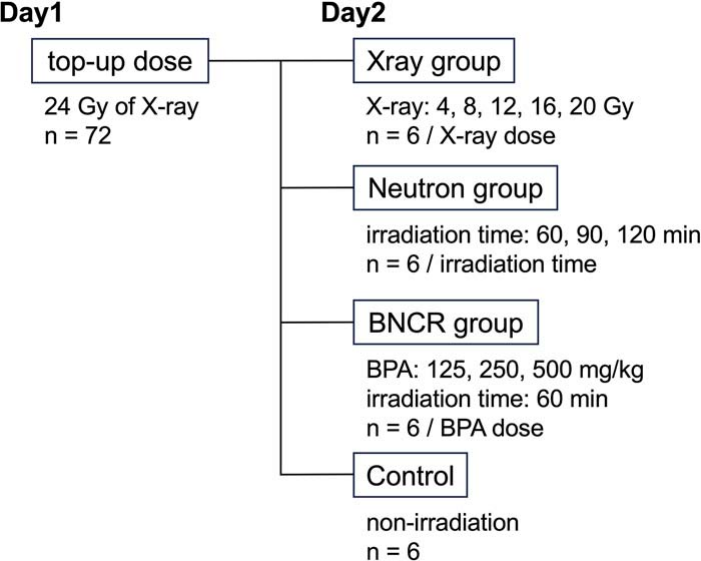
Irradiation design and grouping. Irradiation was performed over two consecutive days: the right hind limbs of all mice were irradiated with a top-up dose of X-rays on Day 1, and X-rays, neutron beams or BNCR were administered on Day 2 (*n* = 6).

Irradiation was performed over two consecutive days as follows because only neutron beam or boron neutron capture reaction (BNCR) could not deliver high doses sufficient to decrease the bone strength: the right hind limbs of all mice were irradiated with a top-up dose of X-rays on Day 1 and X-rays, neutron beams and BNCR were administered to each group of mice on Day 2 (Fig. 1). X-ray and neutron irradiation were performed at Gifu University (PRIMUS Mid-Energy 4 MV linear accelerator, Siemens Healthcare, Malvern, PA, USA) and the heavy water facility at KURNS, respectively.

**Fig. 2:**
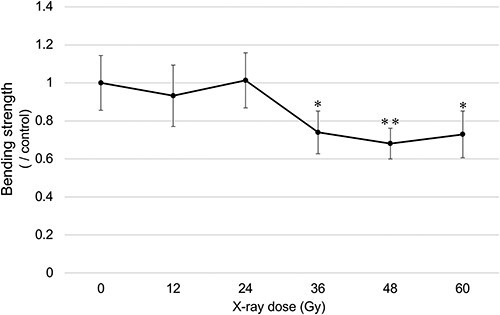
Determination of the top-up dose by X-ray irradiation. The horizontal axis represents the X-ray dose (Gy), and the vertical axis represents the maximum load at fracture (N) obtained by the three-point bending test at 12 weeks after irradiation, which is corrected by the value of the non-irradiated group (*n* = 5). Statistical comparison was performed between the control group and each irradiation group using one-way ANOVAs with *post hoc* Dunnett’s multiple comparison tests (^*^P < 0.05, ^**^P < 0.01).

The top-up dose was determined as follows: mice in each group were fixed to a hand-made jig such that only their right hind limbs were included in the irradiation field and were irradiated at a single X-ray dose of 12, 24, 36, 48 or 60 Gy (five mice for each dose). Bone strength was analyzed 12 weeks after irradiation, and 24 Gy, the maximum dose that did not affect bone strength, was selected (Fig. 2).

To evaluate the changes in bone strength by the boron neutron dose alone, the top-up irradiation time of neutron beam that did not affect bone strength was determined as follows: mice in each group were placed in custom-made chambers and were irradiated for 60, 90 or 120 min at a thermal neutron fluence of 3.10 × 10^12^–6.15 × 10^12^ n/cm^2^ with an output power of 1 MW on Day 2. Bone strength was analyzed 12 weeks after irradiation, and 60-min irradiation was selected for BNCR.

Mice were classified into the following four groups according to the Day 2 irradiation (six mice for each group and irradiation dose): X-ray, five levels of X-ray dose from 4 to 20 Gy in increments of 4 Gy; Neutron, three levels of neutron irradiation for 60, 90 or 120 min; BNCR, neutron irradiation for 60 min, starting 30 min after the administration of BPA at 125, 250 or 500 mg/kg; and Control, non-irradiated except for the top-up dose on Day 1.

#### Bone strength

After tibiae were sampled from each group at 12 weeks post-irradiation, their mechanical properties were measured by the three-point bending tests at diaphysis using MZ-500S (Maruto Instrument Co., Ltd, Tokyo, Japan) as previously reported [7, 16]. In the test, the tibia was stabilized with the lateral side upward at a span length of 10 mm. Then, the load cell was loaded at 500 N from above at the center of the span length with a load rate of 5 mm/min until the fracture occurred. The maximum load at fracture (N), fracture displacement (mm), stiffness (N/mm) and energy to break (N.mm) were determined and calculated relative to those of the Control group. Of these, the maximum load at fracture (N) was analyzed as the bone strength [7].

#### RBE and CBE factors

The decrease in bone strength was set as the biological endpoint in determining the RBE and CBE values, which represent the ratios of the non-boron dose in the Neutron group and the boron neutron dose in the BNCR group required to reach the endpoint to the X-ray dose that causes the same effect, respectively. Since the top-up irradiation time of the neutron beam was determined not to affect the bone strength, the dose-effect curve in the BNCR group, in which the dose was graded only by BPA dose, represents the change by the boron neutron dose alone. Therefore, using the physical dose bone strength graph, the RBE and CBE values were determined from the slope ratio of the Neutron and the BNCR groups to the X-ray group, respectively. The slopes in each group were calculated from the regression lines that were made using the points on the graph where the bone strength was decreasing.

### Statistical analyses

Data were expressed as mean ± standard deviation. The continuous variables, which were the measured values in the boron concentration, were confirmed to follow a normal distribution by a histogram and Shapiro–Wilk test. For comparison between the results of PGA and inductively coupled plasma atomic emission spectrometry (ICP-AES), Student’s *t*-test was used for equal dispersion and Welch’s *t*-test was used for unequal dispersion at corresponding time points in each BPA concentration. To determine the effect of irradiation on bone strength, one-way ANOVAs with *post hoc* Dunnett’s multiple comparison tests were used for comparison between each irradiated group and the Control group. To elucidate the RBE and CBE factors, a regression line was constructed from the physical dose bone strength graph in each group using the single regression analysis, and the 95% confidence interval (CI) of the slope was calculated. Statistical significance was defined as *P* < 0.05. All statistical analyses were performed using JMP pro ver. 16.2.0 software.

## RESULTS

### Boron concentration

**Fig. 3:**
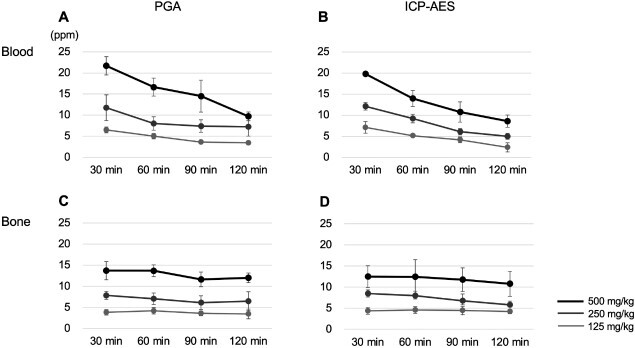
Boron concentrations in the blood and bone measured by PGA and ICP-AES. Boron concentrations in the blood and bone with PGA (**A**, **C**) or ICP-AES (**B**, **D**) at 30, 60, 90 and 120 min after the administration of 125, 250 and 500 mg/kg BPA (*n* = 5). No significant differences are observed between the measurement methods at the time point for each concentration. The horizontal and vertical axes show the elapsed time (min) after BPA administration and boron concentration (ppm), respectively. Statistical analysis was performed using Student’s or Welch’s *t*-test for significant differences between the measurement methods.

The ^10^B concentrations in the blood and tibia after administration of 125, 250 and 500 mg/kg BPA were measured by PGA and ICP-AES, respectively (Fig. 3). Table 1 shows all measurement values in detail, and no significant differences were detected between the PGA and ICP-AES at any time point at all BPA concentrations. Boron concentrations in the blood and bone increased in a dose-dependent manner. While boron concentrations in the blood decreased rapidly by 120 min after administration, those in the bone were kept at the same level from 30 to 120 min after administration. The boron neutron doses required for the subsequent bone strength analysis were calculated based on the mean concentrations in the blood by PGA at 30–90 min after the administration of 125, 250 and 500 mg/kg BPA (Table 2).

**Table 1:** Boron concentrations in the blood and bone measured by PGA and ICP-AES

Time point (min)	Blood (ppm)			Bone (ppm)		
	PGA	ICP-AES	*P*	PGA	ICP-AES	*P*
125 mg/kg BPA						
30	6.50 (0.69)	7.12 (1.38)	0.45	3.81 (0.67)	4.36 (0.83)	0.33
60	5.03 (0.66)	5.13 (0.20)	0.77	4.16 (0.76)	4.55 (0.79)	0.50
90	3.62 (0.32)	4.17 (0.68)	0.18	3.55 (0.54)	4.43 (0.98)	0.15
120	3.46 (0.35)	2.37 (1.09)	0.09	3.41 (1.15)	4.19 (0.51)	0.25
250 mg/kg BPA						
30	11.77 (3.05)	12.12 (0.89)	0.83	7.83 (0.95)	8.46 (0.84)	0.35
60	8.02 (1.60)	9.21 (1.02)	0.24	7.02 (1.36)	7.96 (1.06)	0.31
90	7.42 (1.48)	6.10 (0.75)	0.15	6.09 (1.63)	6.75 (1.59)	0.58
120	7.25 (2.22)	5.00 (0.71)	0.11	6.43 (2.25)	5.78 (0.88)	0.60
500 mg/kg BPA						
30	21.71 (2.17)	19.82 (0.36)	0.16	13.70 (2.17)	12.46 (2.59)	0.48
60	16.65 (2.13)	13.99 (1.90)	0.10	13.66 (1.39)	12.42 (4.09)	0.58
90	14.51 (3.79)	10.77 (2.40)	0.13	11.61 (1.74)	11.75 (2.79)	0.94
120	9.71 (1.03)	8.60 (1.45)	0.25	11.99 (1.14)	10.76 (2.97)	0.46

Values for each parameter are shown as mean (SD). For comparison between values of PGA and ICP-AES, Student’s *t*-test or Welch’s *t*-test was performed at corresponding time points in each BPA concentration (*n* = 5).

**Table 2:** Dosimetry in each group irradiated at KURNS

Group	Irradiation time (min)	BPA administration (mg/kg)	Boron concentration (ppm)	Physical absorbed dose at each component (Gy)			Total physical absorbed dose (Gy)
				Neutron beam dose	Boron dose		
					Dose/ppm	Total dose	
Neutron	60			1.10			1.10
	90			1.55			1.55
	120			2.20			2.20
BNCR	60	125	5.06	1.10	0.26	1.32	2.42
	60	250	9.60	1.10	0.26	2.50	3.60
	60	500	18.11	1.10	0.26	4.71	5.81

### ^10^B biodistribution

**Fig. 4:**
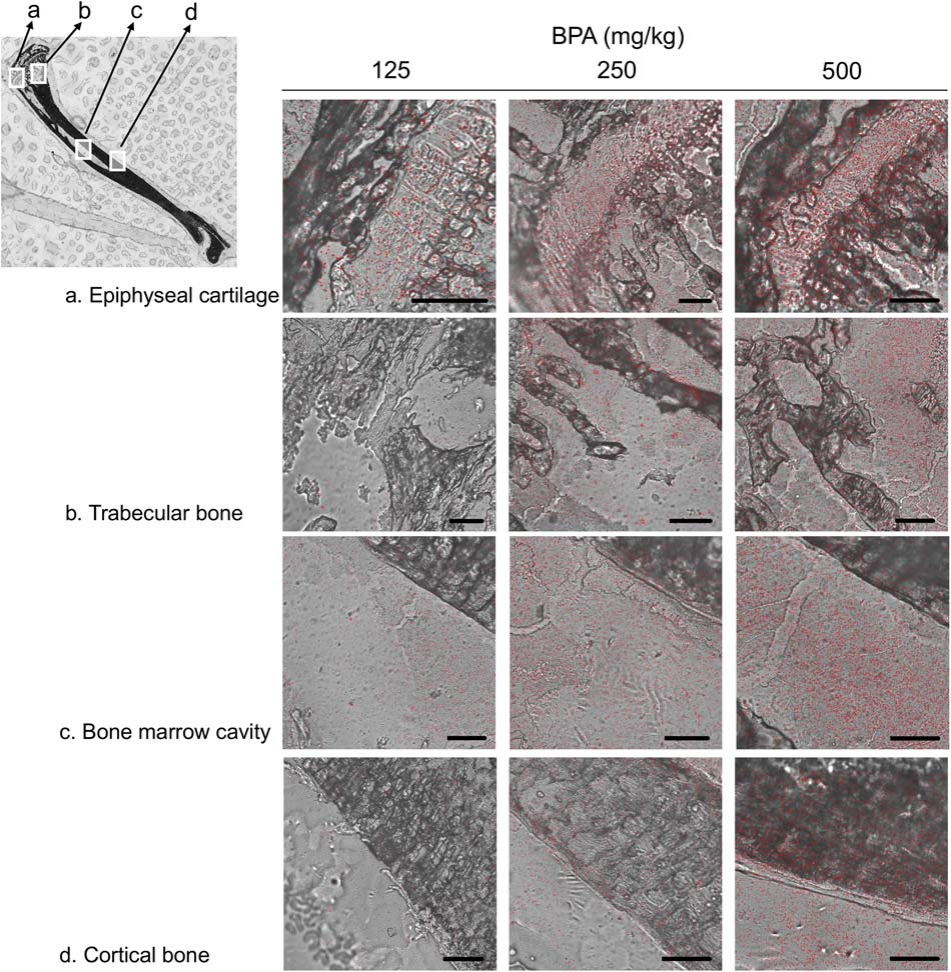
Visualization of ^10^B distribution in the tibia using ARG. Etched pits on CR-39 representing the localization of ^10^B in the epiphyseal cartilage (**A**), trabecular bone (**B**), bone marrow cavity (**C**) and cortical bone (**D**) at 60 min after BPA administration are shown with dots (*n* = 3). Scale bar: 100 μm.

**Fig. 5:**
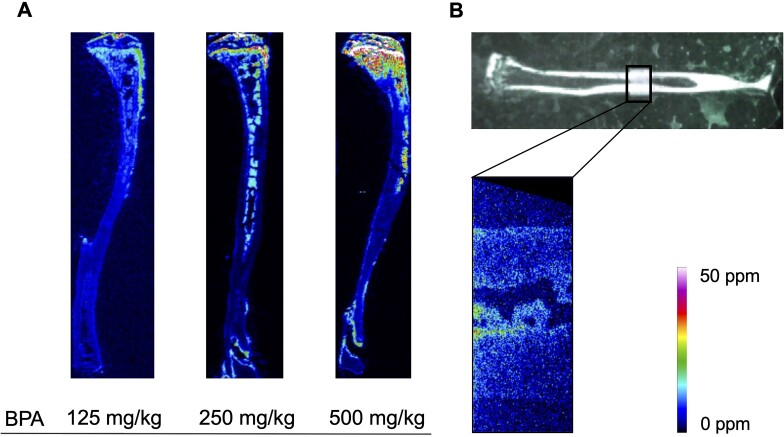
Visualization of ^10^B distribution in the tibia using LA-ICP-MS. Semiquantitative imaging of ^10^B localization in the whole tibia (**A**) and diaphyseal cortical bone (**B**) at 60 min after BPA administration (*n* = 3).

The ^10^B distributions in the tibia 60 min after the administration of 125, 250 and 500 mg/kg BPA were visualized by autoradiography (ARG) and laser ablation ICP mass spectrometry (LA-ICP-MS), respectively (Figs 4 and 5). ARG and LA-ICP-MS visualized the dose-dependent heterogeneous accumulation of administered BPA within the tibia. In both analyses, ^10^B was highly accumulated in the epiphyseal cartilage and trabecular bone of the metaphysis, followed by the bone marrow cavity of the diaphysis. Compared with the higher accumulation in these sites, that in the cortical bone was lower; however, ARG and high-resolution LA-ICP-MS showed some localization in the endosteum and periosteum.

### Bone strength

**Fig. 6:**
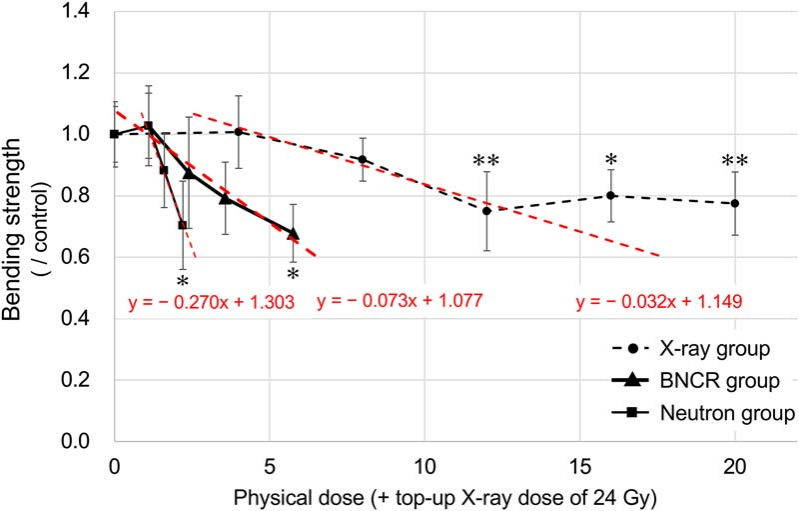
Changes in bone strength in the tibia. The horizontal axis represents the physical dose (Gy) excluding the top-up dose, and the vertical axis represents the maximum load at fracture (N) obtained by the three-point bending test, which is corrected by the value of the Control group. The X-ray, Neutron and BNCR groups are shown as dashed, thin solid and thick solid lines, respectively (*n* = 6). The regression lines in each group are shown as dashed line with their regression equations. Statistical comparison was performed between the Control group and each irradiation group using one-way ANOVAs with *post hoc* Dunnett’s multiple comparison tests (^*^P < 0.05, ^**^P < 0.01).

Twelve weeks after the irradiation of X-ray, neutron beam or BNCR, tibiae in each group were sampled, and the bone strength was measured as an indicator of late effect. The physical doses irradiated to the right hind limbs of mice in the Neutron and BNCR groups at KURNS are shown in Table 2. The mechanical parameters of irradiated tibiae at diaphysis were collected by a three-point bending test (Table 3). The mechanical strength was decreased dose-dependently by up to ~70% at doses of ≥8 Gy in the X-ray group, ≥90 min (i.e. 1.55 Gy) in the Neutron group and ≥125 mg/kg BPA (i.e. 2.42 Gy) in the BNCR group compared with the Control group (Fig. 6). Further, significant changes were observed at doses of ≥12 Gy in the X-ray group (12 and 20 Gy, *P* < 0.01; 16 Gy, *P* < 0.05), 120 min (i.e. 2.20 Gy) in the Neutron group (*P* < 0.05) and 500 mg/kg BPA (i.e. 5.81 Gy) in the BNCR group (*P* < 0.05), respectively. No decrease in bone strength was observed with X-ray irradiation of 4 Gy and with neutron beam irradiation for 60 min (i.e. 1.10 Gy). Although the fracture displacement increased significantly only at 12 and 16 Gy in the X-ray group compared with the Control group, there was no significant difference with the other irradiations. No significant difference in stiffness was observed in any of the irradiation conditions. The energy to break significantly decreased after 90 and 120 min of irradiation in the Neutron group and after 500 mg/kg of BPA in the BNCR group.

**Table 3:** Tibial mechanical analysis by three-point bending test in each group at 12 weeks after irradiation

Group	Maximum load at fracture	Fracture displacement	Stiffness	Energy to break
Control	1 (0.091)	1 (0.185)	1 (0.105)	1 (0.171)
X-ray (Gy)				
4	1.007 (0.118)	1.207 (0.195)	0.926 (0.092)	1.189 (0.122)
8	0.918 (0.070)	1.254 (0.144)	0.964 (0.117)	1.085 (0.147)
12	0.750^**^ (0.129)	1.454^*^ (0.299)	0.852 (0.116)	1.305 (0.247)
16	0.800^*^ (0.085)	1.436^*^ (0.426)	0.834 (0.296)	1.238 (0.348)
20	0.775^**^ (0.103)	1.359 (0.159)	0.921 (0.141)	1.329 (0.248)
Neutron (min)				
60	1.028 (0.130)	0.778 (0.197)	0.982 (0.128)	0.740 (0.220)
90	0.882 (0.121)	0.806 (0.216)	0.761 (0.099)	0.664^*^ (0.126)
120	0.704^*^ (0.144)	0.779 (0.288)	0.991 (0.178)	0.671^*^ (0.133)
BNCR (mg BPA/kg)				
125	0.875 (0.181)	0.738 (0.147)	0.941 (0.264)	0.702 (0.095)
250	0.792 (0.118)	0.854 (0.145)	1.127 (0.491)	0.793 (0.203)
500	0.678^*^ (0.094)	0.825 (0.243)	0.902 (0.507)	0.635^*^ (0.139)

Values for each parameter are shown as the relative values (mean [SD]) compared to that in the control group (*n* = 6). Statistical analysis for each parameter was performed between the control group and each group using one-way ANOVAs with *post hoc* Dunnett’s multiple comparison tests (^*^P < 0.05, ^**^P < 0.01).

### RBE and CBE factors

The straight slopes calculated from each regression line in the physical dose bone strength graph for the X-ray, Neutron and BNCR groups were −0.032 (95% CI, −0.105 to 0.040; *P* = 0.027), −0.268 (95% CI, −0.490 to 0.050; *P* = 0.014), and −0.075 (95% CI, −0.119 to 0.026; *P* = 0.001), respectively (Fig. 6). Based on these results, the RBE and CBE values were calculated to be 8.40 and 2.27 from the slope ratio of the Neutron and the BNCR groups to the X-ray group, respectively.

## DISCUSSION

In this study, we analyzed the biological effects of BNCT on the normal bone by examining the distribution and kinetics of the boron compound BPA in the bone and bone strength following irradiation in healthy mice. Additionally, by determining the response ratio of BNCR to X-ray irradiation, the CBE factor was elucidated when the decrease in tibial strength at diaphysis was set as the endpoint. By conducting the irradiation experiments using this CBE factor and evaluating the late effects on the normal bone, it will be possible to perform BNCT with appropriate doses in the future.

Several studies, to date, have reported the boron concentration measurements in normal whole bone after the administration of boron drug in experimental animals [21–23]. A few studies using PGA have reported that following intraperitoneal administration of the boron compound, sodium mercaptoundecahydro-dodecaborate, the boron concentration decreased by half at 3 h post-dose from its concentration at 1 h post-dose, while the tissue/blood ratio increased 2-fold at 6 h post-dose compared with 2.5 h post-dose [21, 22]. In addition, intraperitoneal administration of BPA maintained the boron concentration in bone up to 3 h after administration [21]. On the other hand, in a study using ICP-AES, when boron concentration was measured every 30 min after the intravenous administration of BPA, it was found to decrease in a stepwise manner until 3 h post-dose [23]. Differences in boron concentrations in the bone between these reports may relate to the differences in the boron compound and route of administration. Furthermore, in the boron concentration analysis of bone marrow after ^18^F-BPA administration, the concentration was observed to decrease rapidly at 2 h after administration, which suggests that boron kinetics may differ depending on the content of bone marrow used for the measurement [24]. In our study, the boron concentration in the normal tibia of mice reached values as low as ~14 ppm even at 500 mg BPA/kg compared with that in the blood. However, the boron concentration in the blood rapidly decreased by 120 min after administration, whereas that in the bone maintained its value in a dose-dependent manner. Additionally, in this study, we compared ICP-AES and PGA results under the same conditions and found no significant differences between these two methods at any dose or time point. This finding emphasizes the accuracy of the values obtained and reliability of the two measurement methods. Another previous study also showed that boron has a strong affinity for bone [25]. In that report, the boron concentrations in the bone and blood were measured by ICP-AES after boric acid was administered to Sprague–Dawley rats. The results showed that boron concentration in the long bones at 5 h after administration remained four to six times higher than that in blood. These findings suggest that the boron neutron dose to the normal bone could be higher with BNCT through a long period of irradiation time because the boron distributed in the bone tends to remain for a certain period compared with that in the blood.

Visualization of ^10^B localization is extremely important because the effects of BNCT on tissue are strongly dependent on the amount and distribution of ^10^B uptake into the tissue. In this study, using the undecalcified serial sections taken from the same mouse, ^10^B mapping in the tibia was performed using two methods, ARG and LA-ICP-MS. Both of these are generally recognized as useful methods for analyzing the ^10^B distribution in soft tissues. Furthermore, the recent establishment of the superposition method with tissue samples has made it possible to detect ^10^B microdistribution; however, few methods are available for hard tissues, such as the bone [26–28]. Recently, a study reported that the use of ARG for hard tissues attained a high correlation with ICP-AES measurements and was useful for measuring boron concentrations [29]. However, to the authors’ knowledge, this is the first study in which ^10^B mapping was performed for the bone. In this study, we found that ^10^B was heterogeneously distributed in the tibia and highly accumulated in the epiphyseal cartilage and trabecular bone at the metaphysis, followed by the bone marrow cavity at the diaphysis. In addition, although the accumulation of ^10^B in cortical bone was poor compared with that in these regions, some accumulation was observed in the endosteum and periosteum. ^10^B mapping showed similar distributions in the two methods. While ARG enabled to observe high-resolution ^10^B distribution in the specific regions of interest, LA-ICP-MS was more versatile because it did not require a neutron source and was suitable for observing the distribution of BPA throughout the tibia as a screening method. Furthermore, LA-ICP-MS can also visualize a specific region of interest in higher resolution; therefore, it is important to adjust the measurement conditions according to the purpose. One of the reasons for the heterogeneous distribution of ^10^B in the tibia is L-type amino acid transporter (LAT1), a membrane protein responsible for the selective cellular uptake of BPA, may be unevenly distributed in the bone tissue. It has been reported that LAT1 is strongly expressed in bone marrow and in osteoblasts and osteoclasts, which are normally present on the bone surface [30, 31]. Therefore, it makes sense that in this study, ^10^B was found to localize to the bone marrow cavity, trabecular bone and periosteum and endosteum of the cortical bone. Although there is no literature that suggests that chondrocytes express LAT1, the abundance of osteoblasts in the perichondrium lining the epiphyseal cartilage and in the distal epiphyseal cartilage during chondral ossification suggests that ^10^B may also be present in the epiphyseal cartilage.

It is generally recognized that bone strength is 70% dependent on the bone mineral density (bone mass) and 30% on the bone quality and that bone structure, accumulation of microdamage, degree of calcification, collagen cross-linking and bone metabolic turnover are the regulations of bone quality [32]. In this study, the bone strength was measured at 12 weeks after irradiation; the strength did not change up to the top-up dose and decreased in a dose-dependent manner in all irradiation groups after irradiation that exceeded the top-up dose. When normal bone is irradiated with X-rays, there is an increase in the number of osteoclasts in the trabecular bone 1–2 weeks after irradiation, followed by a permanent depletion and an increase in the mineral apposition rate in the endosteum up to 4 weeks after irradiation [8]. This results in a decrease in trabecular bone volume and an increase in cortical bone volume in the irradiated bone [7, 33]. This suggests that the modeling phenomenon of increased cortical bone volume compensates for the decrease in bone strength caused by decreased trabecular bone volume. Furthermore, a finding that osteoclasts or their progenitor cells are more radiosensitive than osteoblasts has been reported [34]. These findings support our result that bone strength was maintained after a top-up dose irradiation in this study. The top-up dose of irradiation was performed because only neutron beam or BNCR could not deliver high doses sufficient to decrease the strength. Further irradiation exceeding the top-up dose resulted in a decrease in bone strength, which may be due to the accumulation of microcracks and a decrease in bone mineral density and cortical bone volume caused by the damage of osteoblasts.

Although these reactions are expected to occur in the X-ray group in this study, different changes might have occurred in the BNCR and Neutron groups. In the BNCR group, ^10^B accumulated in the trabecular bone, periosteum and endosteum; therefore, there could be different morphological changes in these regions from those of X-rays. In the Neutron group, the influence by the neutron beam on bone strength was greater, which was reflected in the RBE values. The neutron beams extracted from the reactor used in this study contain a mixture of thermal neutrons, extra-thermal neutrons, fast neutrons and γ-rays. Although no previous study has reported the detailed influence of these radiations on the bone, it is considered that the neutron beams may cause more damage to the bone than X-ray in the regions that affect bone strength. In this study, the physical doses in the Neutron group were graded according to the irradiation times of 60, 90 and 120 min. In the BNCR group, on the other hand, the physical dose was graded by changing the BPA dose while the irradiation time was fixed at 60 min. As a result, the decrease rate in bone strength was higher in the Neutron group than in the BNCR group, which suggests that bone strength is more susceptible to influence to the non-boron dose than to the boron dose.

To evaluate the influence of irradiation with neutron beams and BNCR on bone tissue, pathological and imaging examinations such as micro-CT are necessary. In this study, the decrease in bone strength was set as the biological endpoint; therefore, the top-up dose was too high to investigate the histology and bone morphology because the influence of the top-up dose on these examinations were significant. Conducting a study that uses a neutron beam or BNCR alone without the top-up dose of X-rays would provide a more detailed analysis of the influence. Another limitation of this study is that the observation point was only 12 weeks after irradiation. This was due to the primary endpoint of bone strength decrease; however, changes specific to BNCT, such as early response to irradiation and potential for recovery from damage, may have been overlooked. It is important to analyze the effects of BNCT on normal bone by further investigations that take these points into account.

## Conclusion

This study showed that the administered BPA was found to remain in the tibia of mice for at least 120 min and to accumulate highly in the epiphyseal cartilage and trabecular bone of the metaphysis, followed by the bone marrow cavity of the diaphysis. Furthermore, although BNCR was found to decrease bone strength similar to X-ray irradiation, the effect was less severe than that of the neutron beam. The CBE factor elucidated in this study may be used to determine the appropriate dose for BNCT in future clinical studies.

## Conflict of Interest

The authors declare no conflict of interest.

## Funding

This work was supported by JSPS KAKENHI (JP19K17230).

## Data Availability

The data underlying this article will be shared on reasonable request to the corresponding author.
